# Aortic reinterventions following reoperative frozen elephant trunk aortic arch replacement for the treatment of residual type A aortic dissection

**DOI:** 10.1016/j.xjon.2026.101704

**Published:** 2026-03-04

**Authors:** Joseph Kletzer, Sergey Leontyev, Zara Dietze, Michael Borger, Maximilian Luehr, Christopher Gaisendrees, Tim Berger, Julia Dumfarth, Michael Grimm, Andreas Vötsch, Philipp Krombholz-Reindl, Felix Nagel, Andrea Finster, Martin Czerny, Maximilian Kreibich

**Affiliations:** a Department of Cardiovascular Surgery, Heart Centre, Freiburg University, Faculty of Medicine, Albert Ludwig's University of Freiburg, Freiburg, Germany; b University Department for Cardiac Surgery, Leipzig Heart Center, Leipzig, Germany; c Department of Cardiac Surgery, Heart Center of the University of Cologne, Cologne, Germany; d University Clinic for Cardiac Surgery, Medical University Innsbruck, Innsbruck, Austria; e Department for Cardiac and Thoracic Aortic Surgery, Medical University, Vienna, Austria; f Department of Cardiac Surgery, Paracelesus Medical University, Salzburg, Austria; g Department of Vascular and Endovascular Surgery, Paracelesus Medical University, Salzburg, Austria; h Department of Cardiac Surgery, University Hospital St Pölten, Karl Landsteiner University, St Pölten, Austria

**Keywords:** Frozen elephant trunk, redo surgery, Type A dissection, aortic arch

## Abstract

**Objectives:**

The optimal treatment strategy for residual Type A aortic arch dissections after ascending aortic replacement remains debated. This study evaluated the frequency and outcomes of aortic reinterventions after reoperative total arch replacement with frozen elephant trunk (FET) implantation for residual Type A aortic dissection.

**Methods:**

Between April 2015 and March 2025, 116 patients underwent elective redo arch replacement using the FET technique for residual Type A dissection at 6 European aortic centers. Incidence, procedural characteristics, and outcomes of patients requiring downstream aortic reinterventions were retrospectively analyzed.

**Results:**

In-hospital mortality following FET implantation was 5% (n = 6). Among 110 hospital survivors, 43 patients (39%) required at least 1 aortic reintervention within 48 months: thoracic or abdominal endovascular aortic repair in 36 (80%), graft replacement in 2 (4.4%), and open thoracoabdominal replacement in 4 (9%). No spinal cord injury or disabling stroke occurred postoperatively; 3 patients (6%) died within 30 days of reintervention. After discharge, a second reintervention was necessary in 7 patients (16%), involving endovascular repair in 4 (57%) and open thoracoabdominal replacement in 3 (43%). The cumulative risk of any reintervention was 31.0% (95% CI, 23.1%–40.6%) at 2 years and 55.4% (95% CI, 42.0%–66.9%) at 4 years, whereas overall survival after FET was 87.8% (95% CI, 81.1%-95.1%) and 82.0% (95% CI, 72.1%-93.3%) at 2 and 4 years, respectively.

**Conclusions:**

Reoperative arch replacement with FET yields favorable survival but is accompanied by a substantial need for staged downstream aortic reinterventions, most of which can be managed endovascularly, underscoring the importance of structured long-term imaging surveillance.

**Figure:**
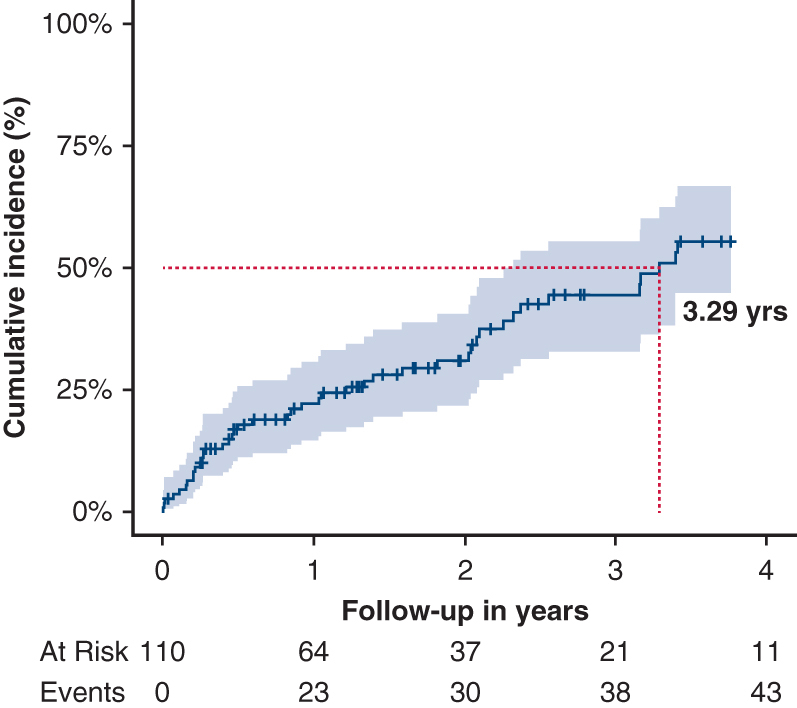
Cumulative incidence of aortic reinterventions following Re-FET after TAAD, with 95% CI.

**Central Message**

Reoperative frozen elephant trunk arch replacement for residual Type A dissection yields good survival but requires frequent staged endovascular reinterventions.

**Perspective**

These findings highlight the need for lifelong imaging surveillance and a planned staged endovascular strategy after redo frozen elephant trunk arch repair.

Ascending aortic replacement, often combined with hemiarch replacement, is the standard intervention for patients presenting with acute Type A aortic dissection.^1^ Despite initial repair, progression of the disease can necessitate subsequent aortic arch reinterventions. Treatment options for these reinterventions include endovascular aortic arch repair and open surgical arch replacement.^2^, ^3^, ^4^ However, endovascular approaches are limited by anatomical constraints, with prior studies showing that after ascending aortic replacement only approximately 70% of patients qualify for secondary endovascular interventions due to factors such as short ascending grafts or graft kinking.^5^^,^^6^ Conversely, open surgical repair, typically performed via frozen elephant trunk (FET) implantation, remains feasible for all clinically operable patients. Despite its widespread use, the optimal strategy between endovascular arch repair and FET for reoperative management is still debated, largely due to a lack of robust comparative randomized data, or even long-term data on the complex multistage treatment strategies often used after FET. This study therefore aimed to assess the incidence and clinical outcomes of aortic reinterventions following reoperative total arch replacement using FET in patients with residual Type A aortic dissection.

## Patients and Methods

The institutional review committee of the University of Freiburg approved this retrospective multicenter study, and the need for informed consent was waived (No. 24-1247-S1-retro; July 30, 2024).

Between April 2015 and March 2025, 116 patients underwent FET total arch replacement following prior hemiarch replacement for Type A aortic dissection at 6 specialized aortic centers: University of Freiburg, Heart Center of the University of Cologne, Medical University Innsbruck, Leipzig Heart Center, Paracelsus Medical University Salzburg, and University Hospital St. Pölten. Of these, 6 patients (5%) died in-hospital and were excluded from further analysis, resulting in a final study cohort of 110 patients. Among these, 45 patients (39%) required aortic reintervention, whereas 65 patients (59%) did not undergo further aortic procedures. The median time from the initial Type A repair to redo FET implantation was 1.67 years (interquartile range, 0.75-3.17 years). Indications for FET were based on current European aortic guidelines, with the decision between open redo FET and endovascular treatment individualized by a multidisciplinary aortic team considering anatomical factors, comorbidities, and patient age.^1^

### Surgical Approach and Technique

All participating centers adhered to a standardized, integrated surgical protocol for the FET technique.^2^ Procedures were conducted via full median sternotomy under hypothermic circulatory arrest with selective cerebral perfusion. Depending on the center, either cardioplegic arrest or the beating heart technique was employed.^7^ Two commercially available grafts were utilized: the Thoraflex (Terumo Aortic) and the E-vita Open/E-vita Neo (Artivion). Indications for distal aortic reintervention after FET implantation were determined on an individual patient basis following current European Association for Cardio-Thoracic Surgery guidelines today.^1^ Reinterventions targeting the downstream descending aorta included either thoracic endovascular aortic repair (TEVAR) or open thoracoabdominal aortic replacement. For TEVAR, the FET stent-graft served as the proximal landing zone usually extending down to the level of the diaphragm. Open thoracoabdominal aortic replacement was performed according to established techniques described previously.^8^^,^^9^ In case of FET graft infection, graft explantation, local debridement followed by orthotopic aortic reconstruction using xenopericardial tube grafts was the treatment of choice as previously reported.^10^

### Data Collection and Definition of Parameters

Data were collected retrospectively relying on the centers’ aortic databases. The modified Rankin Scale (mRS) was used to classify the postoperative stroke severity.^11^ Consulting neurologists evaluated all the strokes. Postoperative strokes causing no clinical symptoms (mRS 0), no significant disability (mRS 1), or slight disability (mRS 2) were classified as nondisabling postoperative strokes.

## Statistical Analysis

Data are presented as absolute and relative frequency or as median (first quartile, third quartile) and are compared between patients with and without an aortic reintervention. A Student *t* test or the Mann-Whitney *U* test were used to compare continuous variables as appropriate. Categorical variables were compared using the χ^2^ test with the calculation of exact values. In case of small group sizes (n < 5), Fisher exact test was used. Survival was analyzed and compared using the Kaplan-Meier method and the log rank test. Cumulative incidence of reoperation was illustrated using cumulative incidence plots with the competing risk of death. Statistical analyses were done using IBM SPSS 21.0 (SPSS Software, IBM Corp) and R software version 4.3.3 (R Foundation for Statistical Computing).

## Results

### Patient Characteristics

Median age of the 110 patients at the time of their Type A aortic dissection was 56.03 years (range, 48.15-64.86 years). At a median (Q1, Q3) age of 61.83 years (55, 69) the FET implantation was performed. Eighty-five percent of the initially implanted FET prostheses had a stent-graft length of 100 mm. Patients were predominantly men (77 patients [70%]), had a high incidence of cardiovascular risk factors, and 15 patients (14%) presented with a heritable thoracic aortic disease. Patient characteristics are summarized in Table 1. There were no statistically significant differences between patients with and without aortic reintervention.

**Table 1:** Baseline characteristics

Characteristic	Overall (N = 110)	Reintervention status		*P* value∗
		No reintervention (n = 65)	Reintervention (n = 45)	
Male sex	77 (70)	44 (68)	33 (73)	.5
Age at FET (y)	61.83 (55.00, 69.00)	63.00 (55.00, 69.00)	59.75 (55.33, 67.22)	.5
Age at Type A (y)	56.03 (48.15, 64.86)	57.81 (50.92, 65.00)	53.61 (46.89, 63.00)	.069
History of smoking	39 (35)	22 (34)	17 (38)	.7
Current smoker	12 (11)	7 (11)	5 (11)	>.9
Hyperlipidemia	47 (43)	27 (42)	20 (44)	.8
History of hypertension	101 (92)	60 (92)	41 (91)	>.9
Insulin-dependent diabetes mellitus	2 (1.8)	2 (3.1)	0 (0)	.5
History of stroke	25 (23)	18 (28)	7 (16)	.14
Dialysis	12 (11)	6 (9.2)	6 (13)	.5
History of COPD	11 (10)	6 (9.2)	5 (11)	.8
History of coronary artery disease	20 (18)	10 (15)	10 (22)	.4
Bicuspid aortic valve	1 (0.9)	1 (1.5)	0 (0)	>.9
Heritable thoracic aortic disease	15 (14)	7 (11)	8 (18)	.3

Values are presented as n (%) or median (Q1, Q3). *FET*, Frozen elephant trunk; *COPD*, chronic obstructive pulmonary disease.

∗ Pearson χ^2^ test, Wilcoxon rank-sum test, or Fisher exact test.

### Aortic Characteristics

The majority of our cohort showed a residual dissection membrane reaching into the brachiocephalic trunk (69%) with no statistical significance when comparing reintervention and non-reintervention groups (*P* = .200). Of all main branches of the thoracic aorta, the right common carotid artery showed the largest heterogeneity between the 2 groups, with 71% being dissected in the reintervention group, whereas only 54% were dissected in the nonreintervention group. Still, this difference did not reach statistical significance (*P* = .068). Residual dissection details are summarized in Table 2 with no statistically significant differences regarding the residual extent of the dissection. Details of prior cardiac and aortic procedures during the initial Type A repair and the FET implantation are summarized in Table E1.

**Table 2:** Aortic details and details of the initial dissection

Characteristic	Overall (N = 110)	Reintervention status		*P* value∗
		No reintervention (n = 65)	Reintervention (n = 45)	
Dissected BCT	76 (69)	42 (65)	34 (76)	.2
Dissected RSA	17 (15)	9 (14)	8 (18)	.6
Dissected RCCA	67 (61)	35 (54)	32 (71)	.068
Dissected LCCA	18 (17)	13 (20)	5 (11)	.2
Dissected LSA	32 (29)	18 (28)	14 (31)	.7
Dissected DTA	94 (87)	56 (8)	38 (84)	.5
Supra-aortic vessel occlusion	2 (1.8)	0 (0)	2 (4.)	.2
Name of occluded vessel				.10
None	31 (94)	22 (100)	9 (82)	
LCCA	1 (3.0)	0 (0)	1 (9.1)	
RCCA	1 (3.0)	0 (0)	1 (9.1)	
Arch abnormality	11 (10)	6 (9.2)	5 (11)	.8
Type of abnormality				.4
None	5 (31)	4 (40)	1 (17)	
Arteria lusoria	1 (6.3)	0 (0)	1 (17)	
Bovine arch	2 (13)	2 (20)	0 (0)	
Isolated left vertebral artery	3 (19)	2 (20)	1 (17)	
LSA before LCCA	1 (6.3)	1 (10)	0 (0)	
Right descending aorta	1 (6.3)	0 (0)	1 (17)	
Truncus bicaroticus	3 (19)	1 (10)	2 (33)	

Values are presented as n (%). *BCT*, Brachiocephalic trunk; *RSA*, right subclavian artery; *RCCA*, right common carotid artery; *LCCA*, left common carotid artery; *LSA*, left subclavian artery; *DTA*, descending thoracic aorta.

∗ Pearson χ^2^ test or Fisher exact test.

### Indications for Reintervention

**Figure 1:**
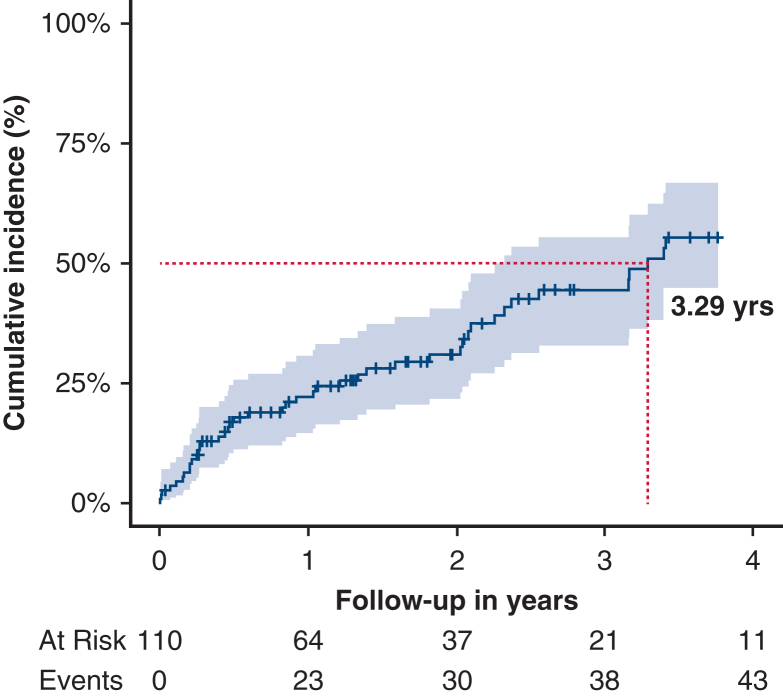
Competing risk regression analysis showing the cumulative incidence of aortic reinterventions following frozen elephant trunk implantation for the treatment of residual type A aortic dissection. The time point where a cumulative incidence of 50% was reached is marked by *dark red dashed lines* and an annotation with the exact time point. The shaded area marks the 95% CI.

The cumulative incidence of aortic reintervention exceeded 50% by the third postoperative year, as demonstrated in Figure 1. Most patients (n = 37 [82%]) were treated for diameter progression and/or the diagnosis of a distal stent-graft induced new entry (dSINE). In 3 patients (7%), the FET stent-graft did not result in adequate true lumen expansion and in 1 patient (2%), the FET stent-graft was introduced into the false lumen. A new thrombus was diagnosed at the distal end of the FET stent-graft in 1 patient (2%) and FET graft infection was diagnosed in 3 patients (7%).

### Reintervention Details

TEVAR was the treatment of choice in 38 patients (84%), 8 (25%) of whom were planned for reintervention prior to FET. In 4 patients (9%) with significant diameter progression of the descending aorta following Type A repair, open thoracoabdominal aortic replacement was planned following FET implantation, 1 (25%) of which was planned beforehand. Lastly, all 3 patients (7%) with FET stent-graft infection were treated with open surgery, graft explantation, local debridement, and orthotopic xenopericardial tube graft replacement of the affected aortic segments accompanied by systemic antibiotic treatment.

### Outcome Characteristics

**Figure 2:**
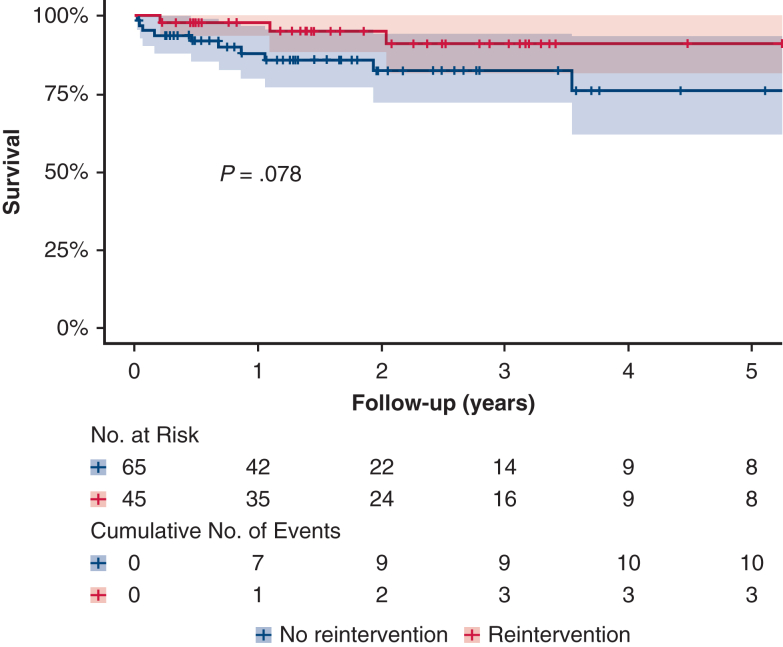
Kaplan-Meier curve of overall survival of patients with and without aortic reinterventions following the frozen elephant trunk (*FET*) technique for the treatment of residual type A aortic dissection. Patients were stratified into those who underwent aortic reintervention after FET. After 3 years, <10 patients are at risk in each stratum. The shaded areas mark the 95% CI.

Outcome characteristics per type of reintervention are listed in Table 3. In-hospital mortality occurred in no patient following TEVAR implantation, in 2 of the 4 patients following open thoracoabdominal aortic replacement and in 1 patient following graft replacement surgery. Also, the incidence of other postoperative morbidity in patients following TEVAR was low with just 2 patients experiencing a nondisabling stroke. Long-term survival (following FET implantation) is depicted in Figure 2 with a nonsignificant difference between patients with and without an aortic reintervention (log-rank test *P* = .078).

**Table 3:** Postoperative outcomes per type of reintervention

Characteristic	TEVAR (N = 38)	TAA replacement (n = 4)	Redo surgery (n = 3)
Temporal spinal cord ischemia	0 (0)	0 (0)	0 (0)
Permanent spinal cord ischemia	0 (0)	0 (0)	0 (0)
Nondisabling stroke	1 (3)	0 (0)	1 (33)
Disabling stroke	0 (0)	0 (0)	0 (0)
New postoperative dialysis	0 (0)	0 (0)	1 (33)
In-hospital death	0 (0)	2 (50)	1 (33)
Aortic reintervention	7 (18)	1 (25)	0 (0)
Planned before FET	8 (21)	1 (25)	0 (0)
Indication in unplanned cases			
Diameter progression	13 (34)	2 (50)	0 (0)
dSINE	9 (23)	0 (0)	0 (0)
Endoleak	3 (8)	1 (25)	0 (0)
Malperfusion	2 (5)	0 (0)	0 (0)
Malposition of FET stent graft	1 (3)	0 (0)	0 (0)
True lumen collapse	1 (3)	0 (0)	0 (0)
Infection	0 (0)	0 (0)	3 (100)
Other	1 (3)	0 (0)	0 (0)

Values are presented as n (%). *TEVAR*, Thoracic endovascular aortic repair; *TAA*, thoracoabdominal; *FET*, frozen elephant trunk; *dSINE*, distal stent graft-induced new entry.

During follow-up, 7 additional aortic reinterventions were performed. In 1 patient, following open thoracoabdominal aortic replacement, pseudoaneurysms were treated with TEVAR. In the TEVAR treatment cohort, 6 patients underwent an additional treatment: 3 patients received further endovascular aortic repair whereas another 3 were treated with open thoracoabdominal aortic replacement.

## Discussion

The principal findings of our study can be summarized as follows. First, reoperative arch replacement with the FET technique is linked to favorable in-hospital and long-term survival, although it has a significant incidence of planned and unplanned secondary aortic reinterventions. Second, although the full surgical spectrum remains essential, most reinterventions can be effectively treated using endovascular techniques, resulting in excellent postprocedural outcomes. Third, patients with residual Type A dissection require vigilant and ongoing aortic surveillance at every stage of their lives.

The cardiovascular risk profile of patients in this study was elevated but consistent with those reported in other investigations assessing long-term outcomes following Type A aortic dissection repair.^12^, ^13^, ^14^ Notably, no statistically significant differences were observed between patients who required aortic reintervention and those who did not. This finding highlights that morphological features of the dissection, such as the location and number of residual communications between true and false lumens, play a more critical role in long-term adverse aortic remodeling than traditional cardiovascular risk factors.

The high cumulative incidence of secondary aortic reinterventions in this study highlights the concept of the FET as a staged treatment option. In fact, retrospectively it is not possible to differentiate between truly unexpected and anticipated, expected, and planned aortic reinterventions. Nevertheless, in patients with significant downstream aortic dilatation of the dissected aortic arch, the FET provides an ideal platform for secondary stent-graft extension as demonstrated before.^15^ However, dSINE remains a common cause for stent-graft extension.^16^ The risk is probably associated with the use of short stent-grafts and a more routine FET anastomosis in zone 2. The 2 factors simplify the procedure and reduce the risk for postoperative spinal cord ischemia but may increase the risk for dSINE formation because of a sharper angle of the FET stent-graft and the descending aorta.^17^^,^^18^ Yet, because dSINE is usually asymptomatic and comparably easy to treat when detected, we believe that the benefit of spinal cord protection outweighs the risk for dSINE formation. In addition, a short stent-graft may potentially not be sufficient to re-expand a true lumen in a chronic setting when the dissection membrane has become thick and fibrous.

In this study, 1 patient was diagnosed with a new postoperative thrombus within the FET stent-graft. The complication has been reported by our group and others and is becoming are more and more significant problem following FET implantation.^19^^,^^20^ The incidence in this collective is extremely low. In fact, studies have suggested that thrombi commonly form in atherosclerotic diseased aortas rather than in dissected aortas and that women are particularly at risk.^19^ After all, in this study, most patients were men. Lastly, in 1 patient the FET stent-graft was implanted into the false lumen. The risk of stent-graft malposition is usually low and mostly described on a case-by-case basis, because it is introduced under direct vision.^21^ Aortoscopy or a wire through the femoral artery may help to ensure true lumen placement.^22^^,^^23^ However, when a large entry tear is present just distally to the left subclavian artery, the stent-graft may shift positions from the true lumen into the false lumen over time. Although malposition in the acutely dissected aorta is associated with extremely bad outcome due to distal malperfusion, in a chronically dissected aorta, distal malperfusion is rare due to the established communications distally.^24^ Fenestration of the dissection membrane and relining of the stent into the true lumen is a reasonable treatment option.

Lastly, 3 patients were diagnosed and treated for aortic graft infection. The complication is rare but higher rates of late infection have been reported in patients with delayed chest closure, a risk that is not uncommon in reoperative aortic arch replacement patients.^25^ Diagnosis is not always simple, but the management of aortic graft infection collaboration (MAGIC) criteria may help in the diagnosis.^26^ We have reported on our integrated surgical management for aortic graft infection previously with acceptable outcome.^10^ In patients with FET graft infection, surgery is complicated by the invasiveness of the procedure. In those patients, we routinely use a hemiclamshell incision for complete removal of the stent-graft component. Others have reported that removal of the stent is feasible through a median sternotomy.^27^ However, through this approach limited access to the descending aorta has to be accepted.

Postoperative outcome in our cohort is generally good. In fact, in patients with graft infection our mortality rate is in line with prior reports and underlines the severity of the disease and the urgent need for preventative measures.^28^ Additionally, in patients needing extensive open thoracoabdominal aortic replacement with an anastomosis to the FET stent-graft we report a 50% mortality rate. Performing the proximal surgical anastomosis up high in the thorax to the FET stent-graft is feasible but difficult and usually requires lung compression resulting in impaired ventilation and a higher risk of pneumonia. Therefore, we have since shifted our surgical treatment algorithm and usually perform open thoracoabdominal surgery after stent-graft extension by TEVAR. This has the benefit of moving the surgical anastomosis site more distally, creating a Crawford Type 4 scenario. Through this stepwise approach we have seen a significant improvement in postoperative outcomes. Possible leakage of the FET stent-graft during secondary thoracoabdominal aortic replacement after graft clamping, with adverse consequences, represents another reason we favor TEVAR extension before proceeding to thoracoabdominal replacement.^29^ Lastly, our study underlines the safety and efficacy of distal stent-graft extension through TEVAR following FET implantation with excellent postoperative results. However, owing to the natural disease progression, addressing the remaining abdominal aorta may become necessary. Open thoracoabdominal aortic replacement remains the cornerstone for the complete aortic fix, but branched and fenestrated endovascular techniques have since emerged as an extremely attractive surgical alternative with promising postoperative results.^30^ The long-term durability in these patients must be evaluated over time.

### Limitations and Strengths

Our study is limited by retrospective nature and the potentially different surgical strategies in the aortic centers. Redo FET surgery followed by downstream aortic reintervention revealed favorable results in these high-volume centers, but these results may not be reproducible in less-experienced centers. However, this investigation contributes valuable knowledge on outcomes after multiple aortic interventions for the treatment of patients with residual type A aortic dissection.

### Data Availability Statement

The datasets presented in this article are not readily available because of the requirements of our institutional review board. Individual reasonable requests will be evaluated by the corresponding author.

## Conclusions

Reoperative arch replacement with the FET technique is associated with good in-hospital and long-term survival. However, it has a significant incidence of planned and unplanned secondary aortic reinterventions. The full surgical spectrum is required to address further aortic repair, but most reinterventions can be effectively treated using endovascular techniques, resulting in excellent postprocedural outcomes. Patients with residual Type A dissection require vigilant and ongoing aortic surveillance at every stage of their life.

## Conflict of Interest Statement

Drs Luehr and Kreibich have received speakers honoraria from Artivion. Dr Voetsch has received travel grants from Terumo Aortic and is a consultant for Edwards Lifesciences. Dr Nagel has received travel grants from Terumo Aortic. Dr Czerny is a consultant for Terumo Aortic and Medira and is a shareholder in TEVAR Ltd and Ascense Medical. All other authors reported no conflicts of interest.

The *Journal* policy requires editors and reviewers to disclose conflicts of interest and to decline handling or reviewing manuscripts for which they may have a conflict of interest. The editors and reviewers of this article have no conflicts of interest.

## Figures and Tables

**Table E1:** Surgical details of the initial surgery, as well as the frozen elephant trunk (FET) after Type A aortic dissection

Characteristic	Overall (N = 110)	Reintervention status		*P* value∗
		No reintervention (n = 65)	Reintervention (n = 45)	
Initial surgery: Type A repair				
Root replacement				
Valve-sparring aortic repair	5 (4.5)	3 (4.6)	2 (4.4)	>.9
Valved conduit	24 (21.5)	9 (14.1)	15 (32.9)	.122
CABG	10 (9.1)	7 (11)	3 (6.7)	.5
Hemiarch	70 (64)	42 (65)	28 (62)	.8
FET				
Additional cardiac procedure	46 (42)	29 (45)	17 (38)	.5
Beating Heart	18 (16)	8 (12)	10 (22)	.2
Zone 2	75 (68)	43 (66)	32 (71)	.6
Zone 3	31 (28)	20 (31)	11 (24)	.5
Extra-anatomic subclavian artery	32 (32)	18 (31)	14 (33)	.8
Name of prothesis				.3
Artivion E-vita	9 (8.2)	7 (11)	2 (4.4)	
Terumo Aortic Thoraflex	101 (92)	58 (89)	43 (96)	
Disabling stoke	7 (6.4)	5 (7.7)	2 (4.4)	.700
Paraplegia, permanent	2 (1.8)	2 (3.1)	0 (0)	.500
ICU stay	7.00 (3.00, 13.00)	7.00 (4.50, 16.00)	5.00 (3.00, 10.00)	.023

Values are presented as n (%) or median (Q1, Q3). *CABG*, Coronary artery bypass grafting; *ICU*, intensive care unit.

∗ Pearson χ^2^ test, Wilcoxon rank-sum test, or Fisher exact test.
